# Effects of trimer repeats on *Psidium guajava* L. gene expression and prospection of functional microsatellite markers

**DOI:** 10.1038/s41598-024-60417-8

**Published:** 2024-04-29

**Authors:** Giovanna Pinto Pires, Vinicius Sartori Fioresi, Drielli Canal, Dener Cezati Canal, Miquéias Fernandes, Otávio José Bernardes Brustolini, Paola de Avelar Carpinetti, Adésio Ferreira, Marcia Flores da Silva Ferreira

**Affiliations:** 1 Centro de Ciências Agrárias e Engenharias, Departamento de Agronomia, Universidade Federal Do Espírito Santo, Alto Universitário, s/n, Alegre, ES 29500-000 Brazil; 2 Laboratório Nacional de Computação Científica (LNCC). Av. Getulio Vargas, 333, Petrópolis, Rio de Janeiro, Quitandinha 25651-076 Brazil

**Keywords:** Trinucleotide repeat expansions, Gene expression, Guava cultivars, SSR, Plant breeding, Genetic diversity, Agricultural genetics, Genetic markers, Genomics, Plant breeding, Plant genetics

## Abstract

Most research on trinucleotide repeats (TRs) focuses on human diseases, with few on the impact of TR expansions on plant gene expression. This work investigates TRs' effect on global gene expression in *Psidium guajava* L., a plant species with widespread distribution and significant relevance in the food, pharmacology, and economics sectors. We analyzed TR-containing coding sequences in 1,107 transcripts from 2,256 genes across root, shoot, young leaf, old leaf, and flower bud tissues of the Brazilian guava cultivars Cortibel RM and Paluma. Structural analysis revealed TR sequences with small repeat numbers (5–9) starting with cytosine or guanine or containing these bases. Functional annotation indicated TR-containing genes' involvement in cellular structures and processes (especially cell membranes and signal recognition), stress response, and resistance. Gene expression analysis showed significant variation, with a subset of highly expressed genes in both cultivars. Differential expression highlighted numerous down-regulated genes in Cortibel RM tissues, but not in Paluma, suggesting interplay between tissues and cultivars. Among 72 differentially expressed genes with TRs, 24 form miRNAs, 13 encode transcription factors, and 11 are associated with transposable elements. In addition, a set of 20 SSR-annotated, transcribed, and differentially expressed genes with TRs was selected as phenotypic markers for Psidium guajava and, potentially for closely related species as well.

## Introduction

Microsatellites are genomic, multiallelic, and codominant molecular markers widely distributed across both the genome of prokaryotes and the nuclear and organellar genome of eukaryotes, consisting of tandemly repeated motifs that range from mono to decanucleotide^1–5^. Hence being called “simple sequence repeats (SSRs)”, or, on occasion, “short tandem repeats (STRs)”^4^. These SSRs are flanked by highly conserved regions, despite their own being hypervariable, due to their inherent high mutation rate of 10^–2^–10^–6^ events per *locus* per generation^1–5^. It is these particulars that make them suitable for genetic profiling^6^, parentage analysis, gene and molecular tagging, linkage mapping, molecular diagnostics, comparative and functional genomics, taxonomic unit identification, analysis of genetic diversity^2,7,8^, and improvement of breeding programs through germplasm characterization^9^.

Polymorphism at SSR *loci* arises from the decrease or increase in repeat number (and as it increases, so does the mutation rate)^2^. There are four mutation dynamics responsible for this variability: replication slippage, unequal crossing over, gene conversion, and retrotransposition^7^. The first two are a consequence of the repetitive sequence’s very tendency to form secondary structures, such as hairpin and triplex, on the DNA strand^3,7,10,11^. During replication, DNA polymerase slippage at hairpins may lead to mispairing bases that escape proofreading and the mismatch repair system, ultimately resulting in the occurrence of deletions or insertions in the repetitive motifs^3,10^. On the other hand, recombination may also culminate in modifications at SSR sites, through unequal crossing over or unequal sister chromatid exchange^2,3,6,7^. As hairpins form over synapsis, one of the homologous chromosomes receives a larger fragment, containing more SSR repeats than the other^7^. As for gene conversion, it is presumed that the substitution of one nucleotide for another (following the model of non-reciprocal exchange) can promote variation in SSR sequences, the same as with minisatellites^7^. At last, retrotransposition may also mediate SSR variability, though its mechanisms have yet to be elucidated^7^.

The number of microsatellites detected in any given organism will depend on the size of its genome. For several different plants, SSR abundance will increase along with genome size, whereas its density will decrease^5^. Ultimately, though, at least one SSR array will be found for every 10 Kb of eukaryotic DNA sequence^12^. But while there is no consensus on a universally most common motif size, triplets (or its multiples) have been well reported as the most frequent SSR class within coding sequences (CDS) in several plant clades, like eudicots, monocots, and less derived plants, so they’re the most likely to turn up, at least in these groups^3,5,13–15^.

The prevalence of trinucleotides in CDS stems from negative selection against frameshift mutations in these regions, from mutation pressure, and positive selection for single amino acid stretch^2,3,15^. Despite that, shorter trinucleotide arrays are favored over longer ones, seeing that the latter may destabilize during meiosis or gametogenesis, and so, seem to experience stronger selection, especially in places where the recombination rate is high^3^. Hence, regardless of their location within coding or non-coding regions, certain tri-arrays exhibit an inherent inability to be conserved for long periods^3^.

Although SSRs in general have once been regarded as neutral markers, it is well known now that they do affect gene expression, be it by epigenetic silencing, by modulating RNA structure and function, or by directly altering the protein sequence^3,16–18^. Either way, they end up assuming functional roles in molecular, cellular, and metabolic processes^1,3,6,7,9,16,18,19^. In fact, several human loci contain trinucleotide repeats, the length of which naturally falls into a typical range^17,20^. But, as it happens in more than forty human neurological disorders^21^, the trimer repetition expands and surpasses the standard motif region length, leading to a pathological state^17,20,21^. Such is the case for fragile X disorders, myotonic dystrophies, and neurodegenerative and polyglutamine diseases ^17,21^. For example, in the most common hereditary genetic ataxia, Friedreich's Ataxia, the untranslated GAA motif repeats more than 66 times, reaching up to 1300 repetitions, when it would normally be repeated 5 to 34 times^6,10,16,17,20–22^^.^

Thus, most scientific investigations focusing on trinucleotide repeat (TR) expansions primarily concerns human diseases. As far as we know, only a few studies address the effect of TR on plant gene expression^22–24^. Thanks to these studies, it is known that the GAA motif, which is normally repeated around 0 to 36 times in the global sampling of the *Arabidopsis thaliana* L. population, is repeated more than 400 times in the third intron of this species’ Bur-0 *IIL1* (isopropyl malate isomerase large sub unit1) gene^23,24^. Homozygous individuals under heat stress (above 27 ºC) or UV-B exposure undergo a growth defect that results in irregularly impaired leaves, the *iil* phenotype^22–24^, implying that environmental conditions do affect gene expression in introns. Carriers of this deleterious genotype do not even progress to the flowering stage^23^. This effect stems from the biogenesis and local accumulation of siRNA, which epigenetically modify the *IIL1* locus through RNA-dependent methylation, down-regulating it^10^. However, one could argue that this same genotype — deleterious within the natural range of *A. thaliana*—could be advantageous, for example, in the Burren environment, where individuals would benefit from delayed flowering on long days, seeing as they would avoid late frosts; posing, therefore, a matter of antagonistic pleiotropy^24^. And indeed, SSRs are related to cryptic genetic variation as well as recent evolution^24,25^. Tabib et al. (2016) even showed how GAA expansion in wild populations has persisted for at least 60 years. Genic regions can therefore be used as microsatellite markers in population studies related to phenotypic variation.

As no previous study has approached yet the effect of TRs in plant CDS, *Psidium guajava* L. (family Myrtaceae) was chosen to further research on this subject. *P. guajava* (2n = 2x = 22)^26^ is a perennial, fruit tree that originated in the American tropic^27–29^, from where it dispersed, reaching wide into the world^27^. Now it is found largely in the tropics and subtropics, where its fruit—guava—is produced mainly by countries like India, China, Indonesia, Thailand, Pakistan, Mexico, and Brazil, respectively^27,30^. Besides its fruit, each and every part of its anatomy is exploitable in favor of its countless active principles. For example, its roots, stem, bark, and leaves are also rich in antiseptic, anti-bacterial, anti-inflammatory, antihypertension, diuretic, astringent, antispasmodic, cough sedative, antidiarrheic, anti-obesity, antitumor, anticancer, and cytotoxic proprieties, thus being capable of treating maladies of the gastrointestinal and respiratory tracts, skin, and wounds. Other compounds also treat diabetes, rheumatism, and general^9,31–35^. This vast herbal application is based on the myriad of secondary metabolites produced by this species^31,33–35^, which evidence its adaptation to disparate biogeographic conditions. On the other hand, its leaf isolates serve as the reducing agent for the green synthesis of iron magnetic nanoparticles^36^, which are of ample value to nanobiotechnology, being lately employed in oil spill remediation^37^, biodiesel synthesis^38^, protein loading^39^, as well as detection, imaging, and targeting of tumors^40^.

So, this work aims to investigate the effects of trimer repeats on the global gene expression of P. guajava, focusing on two genetically divergent Brazilian cultivars, namely Cortibel RM and Paluma. The Paluma cultivar is the most widespread in Brazil, characterized by a robust genotype, with easy propagation^41^. Concurrently, the cultivation of Cortibel guava has increased in the Brazilian Southwest because of its high productivity, and extended shelf life, making them suitable for export^42^.

Transcript levels and differentially expressed genes containing trimer repeats were examined across five distinct tissues (roots, shoots, young leaves, old leaves, and flower buds) in the Paluma and Cortibel RM using Illumina RNA-Seq. Trimer repeats were characterized considering both their size and the Gene Ontology annotations associated with their genomic loci. Among these predicted genes, those exhibiting greater stability and variability in terms of gene expression were selected to develop trinucleotide microsatellite markers involved with potential phenotypic changes. The resultant markers will subsequently be employed to validate the observed differential gene expression patterns.

## Methods

### Plant material, rna extraction, transcriptome assembly and alignment

The seedlings grew in nurseries. Tissues from two Brazilian cultivars, Cortibel RM and Paluma, comprising root, shoot, young leaf, and old leaf, were collected after a growth period of one hundred days. At the time of sampling, only Cortibel RM exhibited the presence of flower bud tissue. Consequently, this specific tissue was not obtained from Paluma. The twelve most uniform plants of each cultivar were selected by their morphological characteristics, as to ensure sample homogeneity. Four samples were collected from each of the above-mentioned tissues and mixed in equal proportion to form two pools of each cultivar. The total RNA was extracted using the CTAB method, chosen for its efficiency in yielding high-quality RNA across various genotypes, tissues, and stress conditions of P. guajava^43^.

The quality of total extracted RNA was measured with TapeStation System (Agilent), and the quantity with Qubit (Thermo Fisher Scientific). Samples with an RNA Integrity Number (RIN) greater than 5 were considered to be of high quality44.

The high-quality RNA was further subjected to rRNA removal using the RiboMinus Plant kit (Thermo Fisher Scientific). Libraries were prepared using Illumina TruSeq RNA Library Prep Kit, following the manufacturer's guide. Quantification was done with Qubit (Thermo Fisher Scientific), and fragment sizes were estimated using the TapeStation System (Agilent).

We sequenced two libraries from each tissue with Illumina Nextseq500 in Midi mode, generating paired-end reads of 75 bp. In the end, there were 18 libraries. The Illumina FASTQ files were analyzed with the NGS QC Toolkit software^45^ to check the quality of the sequenced reads, as well as the presence of artifacts that stem from the preparation of the sequenced samples, such as adapters and low-quality sequences, or from the sequencing itself.

The sequences were assembled de novo with Trinity Galaxy (version 2.15.1)^46,47^, where the sequences were grouped into contigs and singlets. The annotation was done by comparison with plant amino acid sequence databases. The contigs were also compared using the curated RefSeq^48^, Swiss-Prot^49^, TrEMBL^49^, and NCBI amino acid sequence^50^ databases. We processed the assembly and annotation process with the Galaxy instance of the Autralia platform (https://usegalaxy.org.au/). The best resulting alignment was selected to annotate the putative transcripts.

The eighteen RNA-Seq libraries were filtered, then mapped with BWA-MEM (Galaxy version 2.2.1) to the *P. guajava*’s draft genome, which is under submission. This alignment's read counts were used in PCA, relative abundance, and differential expression analyses.

### Expression profile analysis of the RNA-seq data

Differential expression analysis requires the preprocessing of RNA-Seq data through filtering and alignment, for this purpose we used DESeq2::deseq()57 within R (Core Team, 2022). This methodology is based on the negative binomial distribution, applied to calculate the variation in transcript expression among different samples, as well as its statistical significance. Thus, the comparisons were made at the tissue level, between cultivars, arbitrarily choosing to use Cortibel RM as control and Paluma as treatment. Only genes present in both biological repeats were accepted, as to ensure data processing quality. Genes were considered differentially expressed when one duplicate of a cultivar displayed a significantly different mean from the other by the Student’s t-test (p-value < 0.05), being two times higher or lower (fold-change) than the other. We applied the FDR (False Discovery Rate) statistical test58 on the statistical significance of gene expression values between samples to filter out false positives.

Read counts underwent Z normalization, and the new Z values served as input to Principal Component Analysis (PCA), using R software v.0.55 (Core Team, 2022) with the packages::functions stats::prcomp^59^ and plotted using ggbiplot::ggbiplot^60^. A Ward-MLM (Modified Location Model) and UPGMA (Unweighted Pair Group Method with Arithmetic Mean) clusters were also generated using gplots::heatmap.2^61^ for the relative abundance analysis while we used the package::heatmaply::heatmaply() function^85^ for the differential expression profiles.

### Mining of SSRS, functional annotation, and enrichment analysis

**Figure 1:**
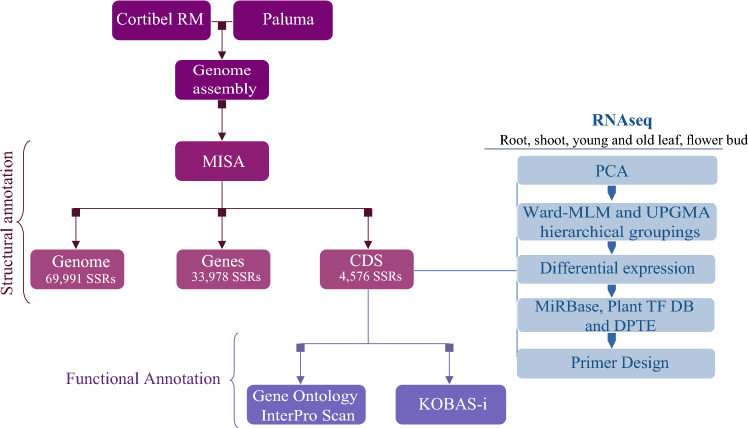
Overview of the methodology implemented in this study. Initially, we employed de novo assembly techniques to construct a hybrid draft genome using the Cortibel RM and Paluma cultivars. Subsequently, the MISA tool was utilized to identify microsatellites within the genome, gene, and coding sequences, resulting in the detection of 69,991 SSR, 33,978 SSR, and 4576 SSR, respectively. Functional annotation of the coding sequences was performed using Gene Ontology and InterPro Scan. Additionally, an enrichment analysis was conducted using KOBAS-i to determine the functional profile of the coding sequences. RNA-Seq data (read counts) of the CDS with TR from the root, shoot, young leaf, old leaf, and flower bud of both cultivars’ tissues served as input to PCA, relative abundance, and differential expression analysis. We identified microRNA (miRNA), transcription factors (TFs), and transposable elements (TEs) in genes with triplet repeats on the miRBase, Plant TF DB, and DPTE databases, respectively. Primers were then designed for the differentially expressed and upregulated genes that had functional annotation, but also for those in which the motif repeat number was unusually high (equal to or above 11), or for those in which the expression level was significantly high (equal to or above 5) in all tissues at the same time.

The genome, genes, and CDS were mined for SSR with MISA5,62. The parameters were set to default as follows: monomers ≥ 10, dimers ≥ 6, while trimers, tetramers, pentamers, and hexamers ≥ 5. A Python script assigned gene ontology identifiers to the genes containing trimer repeats, based on results from the InterPro63 and eggNOG64 databases. We retrieved the GO terms and their enrichment analysis with GOSlimViewer65 and KOBAS-i66, using this tool's default parameters. All charts except for those of enrichment were built in R Studio v 4.2.1. The flow chart in Fig. 1 depicts the summary of our methodology.

### Criteria for putative gene selection and primer design

Among the entirety dataset of genes containing triplet repeats, a filtering process was employed, focusing on those demonstrating transcription with Z-values (normalized read counts) equal to or greater 1 in any tissue of both Cortibel RM and Paluma. Subsequently, the selection was refined to encompass differentially expressed genes that exhibited upregulation and had been annotated by either Gene Ontology (GO) 67,68 or InterProScan69. We also prioritized genes possessing an unusually high Z value (z >  = 5) in all tissues at once, as well as those in which the motif’s repeat number was notably large (RN > 11).

Primers for coding sequences with TR were designed in NCBI PrimerBLAST70, avoiding annealing at exon-junction regions. The design specifications were: guanidine-cytosine content of 50–60%, melting temperature (MT) of 60–62 ºC, primer size of 20 to 22 nucleotides, and amplicon length of 110 to 180 base pairs. Hairpins and dimers were evaluated by IDT OligoAnalyzer71. Primers with ΔG values between -3 kcal/mol and -9 kcal/mol were considered the best primers.

The genes were amplified by PCR in 20 μL reactions using GoTaq® DNA.

Polymerase (Promega, USA), following the manufacturer's instructions.

### Ethical approval

This article does not contain any studies with human participants or animals performed by any of the authors.

## Results and discussion

**Figure 2:**
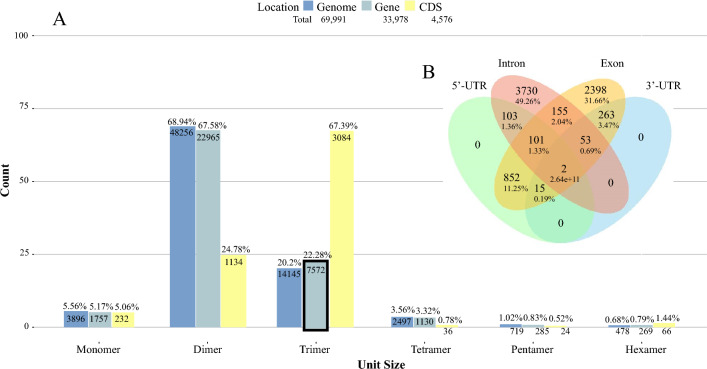
Structural annotation of the SSRs detected by MISA within the Psidium guajava genome. (**A**) Amount of SSRs detected in the genome, genes, and coding sequences of Psidium guajava, arranged by motif size. A total of 69,991 SSRs were identified in the genome (dark blue), 33,978 SSR in genes (light blue), and 4.576 SSRs in CDS (yellow). Out of the 7572 trinucleotides found in genic regions, 3084 occur in CDS (i.e., exons). (**B**) Region and number of trinucleotides within 6213 genes (a total of 7572 trinucleotides were detected). The proportion of trimers found in each genetic region is calculated by dividing the number of trimers found in each region by the total number of trimers detected (e.g.: 3730 ÷ 7572 = 49.23%).

We opted to mine the genome, genes, and coding sequences for motifs consisting of monomers up to hexamers. The result is shown in Fig. 2A, where the two most represented classes of SSRs — dimers and trimers—are evident (Supplementary Table S1). Our results corroborate with previous descriptions^3^, in which dimers and their multiples are identified as the most frequent in non-coding sequences, and trimers and their multiples as the most frequent in CDS. Here, however, only dimers (but not their multiples) have high frequencies in genic and intergenic regions, and only trimmers (but not their multiples) display high frequencies in CDS. Since this work aims to develop putative markers for phenotypic variation, we stuck to the coding sequences and specifically, we concentrated our efforts on exploring the trinucleotide repeats within the coding sequences, as this SSR class represents the most abundant category.

The Venn Diagram (Fig. 2B) demonstrates that a higher proportion of triplets are exclusively observed within intronic regions (49.23%) compared to any other genomic region. Exons have the second highest occurrence (31.65%) of trinucleotides in gene regions, followed by the 5'-UTR promoter region (12.8%). A small percentage of trinucleotides occur in 3'-UTR regions (4.39%), and an even smaller percentage in intron–exon junctions (4.1%). No trinucleotide occurs only upstream or downstream of introns and exons.

Although at first glance Fig. 2B does not seem to support the results found in Fig. 2A, since there seem to be more triplets in introns than in exons, one should consider the longer length or higher frequency of introns than exons in a genome^72^. When evaluating the intron/exon ratio, the larger density of trimers in exons stands out, sometimes containing more than a single motif within a sequence. As mentioned earlier, the frequency of triplets (and their multiples) is higher regarding other SSR classes in CDS due to selective pressure against frameshift or the stretching of some specific amino acid in the protein.

**Figure 3:**
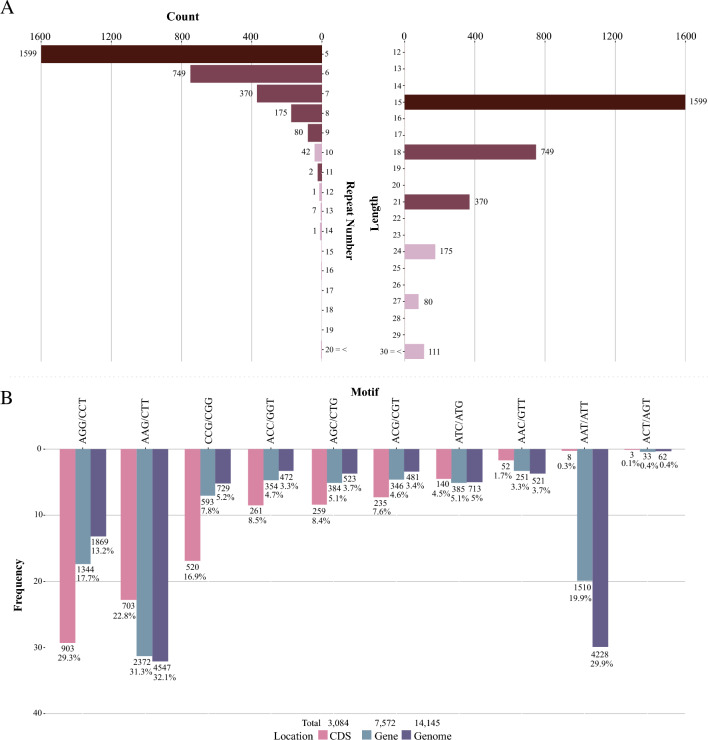
Structural annotation of trimer motifs in guava CDS. (**A**) Absolute frequencies of the number of repeats (left) and trimer motif region lengths in CDS. (**B**) Occurrence number of the most represented trimer motifs in CDS.

The smallest repeat numbers and motif lengths of triplet microsatellites are the most common (Fig. 3A; Supplementary Table S2). Especially in exons, since intronic sequences are more likely to carry longer repeats than CDS (Supplementary Table S1, S3). In *A. thaliana*, less than 1% of triplets exceed 5 repeats, with no expressed gene containing more than 41 repeats^23^. Also, the Bur-0 genotype, which features trinucleotide expansion, is rare among *A. thaliana* strains^23^. Low repeat numbers are more common, probably because short microsatellites are the starting point for subsequent extension of their length^5^. They are also more stable than long SSRs^3^, which are more prone to mutation, gaining repeats faster than shorter arrays. Natural selection also acts against repetitive sequence growth^2,3,73^, resulting in long SSR sequences also being more prone to losing repeats than shorter SSRs^2^.

In Fig. 3B, we see the top ten occurring motifs in CDS (Supplementary Table S3, S4). Among them, the five most frequent ones (AGG, AAG, CCG, AGC, and ACC, respectively) have at least one guanine or cytosine. GC content is always more enriched in genic regions, and indeed, GC content in this draft genome was 39.49%, while in CDS it was 49.68%—a measure similar to that of other eudicots. In addition, the frequency of these bases is closely related to epigenetic control via methylation^74^. Eimer et al.^22^ demonstrated in their experiment, where the accumulation of siRNAs and subsequent RNA-dependent methylation of the IIL1 locus caused the downregulation of this gene, setting off the *iil* phenotype. As shown later, several of the 72 differentially expressed genes from our gene set are downregulated in at least one tissue.

Moreover, the motifs above-mentioned encode amino acids that, when present in the primary structure of a protein, cause drastic changes in its native conformation. The AGG and AAG motifs, for example, encode positively charged amino acids (arginine and lysine, respectively) that result in electrical attraction or repulsion in the protein's three-dimensional structure^75^. This is worth noting because electrostatic interactions play multiple roles in protein-RNA complexes, such as the formation of the initial complex and its maintenance^75,76^. Arginine, in particular, is an effective suppressor of protein interactions^77^. The CCG motif, on the other hand, encodes proline, an amino acid that produces disruption in the secondary structure whenever present^78^. The AGC and ACC motifs encode serine and threonine, respectively—two polar, uncharged amino acids that can affect protein stability^79^.

When the genes harboring trimer repeats were transcribed, the expression values ended up centering mostly around motifs beginning with cytosine or guanine, with the motifs containing these bases displaying the highest means (Supplementary Figure S1-A). Likewise, the genes with the lowest repeat numbers (5–9) were the most expressed (Figure S1-B). On one hand, this would be expected from SSRs in coding sequences since these are conserved regions and the lower inherent mutation rate of shorter microsatellites means more stability in its region length. On the other hand, high level expression of a gene containing long SSR lengths is rare. The literature also points to highly repeated motifs being accompanied by low expression, as is the case of *A. thaliana* Bur-0 strain’s *iil* phenotype and Friedriech's ataxia^22^. Despite this, repeat numbers 16 and 17 display higher means than the smallest ones, where most of the expression data concentrated (Figure S1-B). In humans, large AC repeats in the *NOS1* gene promoter lead to high expression levels, with shorter AC lengths being associated with impulsive behavior^7^. Long lengths of composed SSR sequences of poly-AC and poly-AG upstream of the transcription start site of the *PAX6* human gene induce high expression levels, which is associated with myopia^7^. In our dataset, the 5'-UTR promoter region has the third-highest frequency of triplets in genes (Fig. 2B). Further exploration of this data would be required after Srivastava et al. (2019)^15^ pointed out that the preference for the accumulation of a particular class of long repeats may indicate selective pressure on these elements in an organism-specific manner.

**Figure 4:**
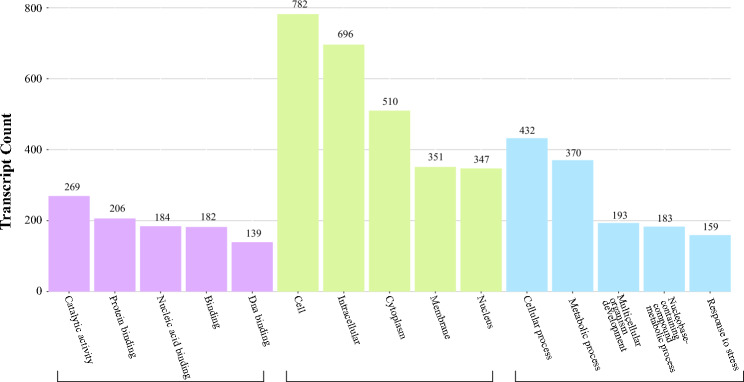
Gene Ontology functional annotation of the 1,107 transcribed genes containing trinucleotide.

**Figure 5:**
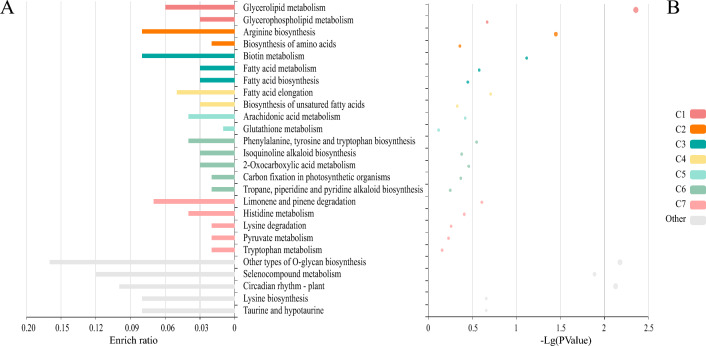
Functional profile ofthe 1,107 transcribed genes carrying trimer repeats by KOBAS-ienrichment analysis, using Eucalyptus grandis genome as a reference (the closest relative of P. guajava^84^).

If the largest part of this dataset refers to slightly repeating trimer SSR sequences, then Fig. 4 shows their gene ontology related to molecular function, cellular component and biological process. Cellular, intracellular, and cytoplasmic structures are not only the most abundant GO terms, but also the most represented, judging from their enrichment ratio in Fig. 5A and the relevance of the glycerolipid and glycerophospholipid metabolism clusters (the groups of overrepresented genes^66^) in Fig. 5B.

SSRs. A) Number of genes with trimer repeats associated with molecular functions. B) Number of genes with trimer repeats associated with cellular components. C) Number of genes with trimer repeats associated with biological processes.

A) Clusters of statistically significant functional terms associated with the transcripts containing trimer repeats and their enrichment ratio. B) Bubble plot of the scattering of p-values for each cluster and their functional terms. The node size, from small to large, represents the six levels of p-value (or R scores) — 0, 0.5, 1, 1.5, 2, 2.5^66^.

**Figure 6:**
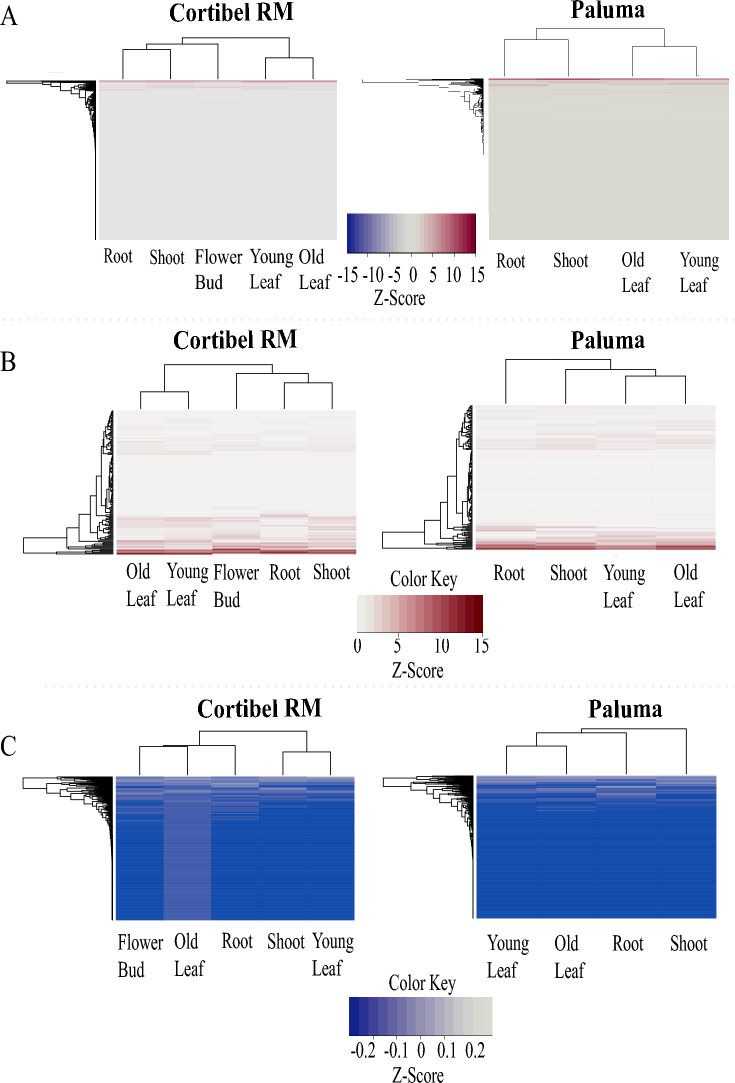
Ward-MLM and UPGMA hierarchical groupings of the normalized read counts of the 1,107 transcribed genes carrying triplet SSRs from the tissues root, shoot, young and old leaves, and flower bud of Cortibel RM and Paluma cultivars. (**A**) Relative abundance of all 1,107 transcribed genes with TRs. (**B**) Relative abundance of the 142 upregulated (z-score > 1) genes with TRs. (**C**) Relative abundance of the 811 downregulated (0,5 < z-score < 0) genes with TRs.

**Figure 7:**
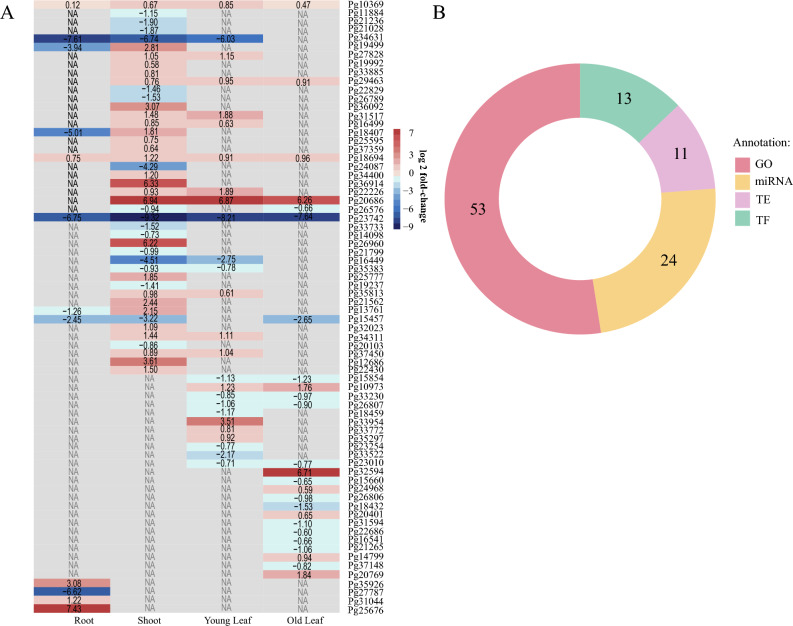
Differential expression of the 72 genes with trimer repeats from the roots, shoots, young and old leaves* of Cortibel RM (control) and Paluma (treatment) cultivars. (**A**) Differentially expressed genes harboring triplet SSRs from the roots, shoots, young and old leaves of both cultivars.The darker the blue, the more downregulated the gene is, and the redder the red, the more upregulated the gene is. (**B**) Amount of functionally annotated differentially expressed genes associated with transcription factors (TF), micro.

The principal coordinates analysis (PCA) of transcribed genes carrying triplet SSRs in Supplementary Figure S2 shows the similarity between the experimental replicates, as well as the differences between the tissues in their expression profiles. These differences are further validated in the relative abundance analysis (Fig. 6A:C; Supplementary Table S5, S6). There is a large variation in gene expression ranging from -15 to 15 *z*-values, although most of the data centers around − 0.5 to 0.5 *z*-value (Fig. 7-A). A small set of genes is highly expressed, independent of the cultivar (Fig. 6A). Thus, the discrepancy in the read counts normalized to *z*-values is so high that the few upregulated genes end up masking the expression level of the others (Fig. 6A). These upregulated genes also appear to be highly expressed in all tissues (Fig. 6B), although one group of genes, in particular, is highly expressed only in Cortibel RM's flower bud and no other tissue. The chlorophyllated tissues usually cluster together, except when looking at Cortibel RM downregulated genes (Fig. 6C), whose effect can be seen in old leaves, where most genes are more expressed than other tissues. In addition, Cortibel RM downregulated root genes are clustered with the leaf tissue in Fig. 6C. These same effects are not observed in Paluma. Overall, we can see that the expression profiles and clusters are similar, but not identical. There is an effect of genes with trimer repeats between tissues and cultivars.

This effect can be further observed in Fig. 7A, the heatmap of differential expression between tissues of Paluma, as opposed to Cortibel RM. Genes such as Pg18407, Pg19499, Pg23742, Pg24087, Pg27787, and Pg34631 are much less expressed in Paluma than in Cortibel RM. These genes are related to sulfate transport and the EF-hand1 calcium-binding site, iron-sulfur cluster, UDP-Glycosyltransferase, sulfate transport and GTPase, and the B3 DNA-binding domain. Pg16449 is another gene that seems more downregulated in Paluma than in Cortibel RM, but it hasn’t been annotated either by GO or InterProScan. On the other hand, the genes Pg20686, Pg25676, Pg26960, and Pg36914 are much more expressed in Paluma than in Cortibel RM, being associated with RNA recognition and binding, dirigent proteins, aldehyde dehydrogenase and potassium ion transport, and cobalamin-independent methionine synthase, respectively. Though the Pg32594 gene is also much more upregulated in Paluma than in Cortibel RM, no annotation was found by GO or InterProScan.

Figure 7A also shows that shoots are the tissues with the highest number of differentially expressed genes containing trimer repeats, having the most variable expression levels as well. This was to be expected, considering that shoots are tissues undergoing differentiation. In contrast, we have the root as the tissue with the least number of differentially expressed genes but showing quite a difference in their expression. Young and old leaves have a close number of genes with differential expression.

The transcribed genes sum is 1,107 (from a total of 2,256 harboring TRs), of which 72 are differentially expressed (Suplementary Table 8). The Fig. 7-B summarizes the count of microRNAs, transcription factors, and transposable elements (Supplementary Table S7). Twenty-four were annotated by miRBase as stem-loop (also known as hairpin or hairpin loop) forming pre-miRNA — secondary structures on single strands of RNA formed by regions with complementary sequences when read from opposite sides —, very commonly found in microsatellite sites. Thirteen, annotated by the Plant TF database, encode transcription factors, involved with the ARF family proteins, bHLH, B3, DBB ERF, HD-ZIP, MYB, Trihelix, WRKY, prothodermal factor 2, ethylene-responsive element binding factor, telomere repeat binding factor 1, auxin response factor 9, B-box zinc finger protein with CCT domain, and with the NF-X-like 1, a gene whose product functions as a negative regulator of the defense response induced by trichothecene phytotoxins^83^. In fact, when analyzing the annotations of the entire gene set (2,256) as a whole, it is clear that many are resistance or stress response genes (which goes back to Fig. 4). Lastly, the remaining eleven differentially expressed genes were annotated by the Dioecious Plants Transposable Elements database (DPTEdb), with eight of them being helitron transposons and three being retrotransposons — two long terminal repeats (LTRs) and one long interspersed nuclear element (LINE).

RNAs (miRNA), and transposable elements (TE). *Since flower bud RNA was extracted for Cortibel RM alone, such tissue wasn't included in this analysis for comparison reasons.

In short, MISA identified 3,084 trinucleotides in 2,256 genes (Supplementary Table S8). Less than half are transcribed (1,107). But among them, only 72 are differentially expressed. Thirty-nine of these are upregulated, but only twenty-three have been annotated either by Gene Ontology or InterProScan. Some other genes with trimer repeats have large repeat numbers (NR; genes Pg11715, Pg32078, Pg47442, Pg47443, Pg50279) or very high normalized expression levels (EL >  = 5) in all tissues simultaneously (genes Pg19110, Pg21266). So, primers were designed for 20 genes, of which the relation of gene ID, SSR motif, primers, and functional annotation is shown in Table 1. Finally, polymorphisms were identified in the Paluma and Cortibel RM gDNA, indicating putative functional markers for the species (Supplementary Figure S3). These results highlight the potential for development of functional SSR markers for P. guajava.

**Table 1:** Forward and reverse primers for differentially expressed and upregulated genes with trimer repeats (or for those with unusually high RN or EL), plus gene functional annotation. MT F, AT F,MT R, and AT R refer to the melting and annealing temperatures of each primer, forward and reverse, respectively. We used the melting temperature as the annealing temperature of each primer.

Gene ID	Motif	Primer F Primer R	MT F | MT R (ºC)	Amplicon gDNA	Amplicon cDNA	Functional Annotation
Pg14098	(TTG)_5_	5’ AGGGCCTTTCTGCTGATGAC 3’ 5’ GGCATAGGGCACGTTCTAAAG 3’	61,7 | 61	145	145	Homeobox-leucine zipper protein GLABRA 2-like
Pg15660	^1^(GAC)_6_ ^2^(CGG)_6_	5’GTTCGCACGCGATTCAGAC 3’ 5’ ACATCAGCTTCTGCCTCACC 3’ 5’ AGCCGTGGAATCTGAGGAC 3’ 5’ CGACTTCGTGTCGTTTATACCG 3’	63 | 60,4 60,2 | 61,8	128 137	128 137	Protein of unknown function (DUF1639)
Pg16499	(TCT)_5_	5’ GAAGTTCGCCCACGATCTTATC 3’ 5’ CGGGTTCTTGAAGAGGGAAA 3’	62,2 | 61,5	100	100	Conserved site Chaperone DnaJ
Pg16451	(CGC)_5_	5’ ATGTACGCCATGGGGAAAG 3’ 5’ CTCTTCTGCCACAGCACGA 3’	60,7 | 61,4	146	146	Protein of unknown function (DUF620)
Pg19499	(CCT)_5_	5’ TACCTCCTCACCCGCGCT 3’ 5’ GCCGGCGAACTCATCGGTGAT 3’	61,3 | 62,3	119	119	F1/V1/A1 complex, alpha/beta subunit, N-terminal domain, superfamily ATPase
Pg20103	(CCG)_5_	5’ GTACGGAATATCAGCGGCAAC 3’ 5’ GGAAGGAAGGGAGAAATAGGG 3’	61,7 | 60,3	133	133	Homeobox-leucine zipper protein
Pg20401	(AGC)_5_	5’ ACAGCTCGTCCTCGTCGTC 3’ 5’ ATCGCGAGCTCTCTTCTCG 3’	61,6 | 60,9	107	107	Kinesin-like protein Tesmin/TSO1-like CXC domain
Pg21028	(GTC)_5_	5’ CATCCGCCAGCATCTTCTC 3’ 5’ ACGAAGACGTGTCGAAGCTC 3’	61,9 | 60,6	107	107	DNA-binding domain superfamily AP2/ERF
Pg21266	(CTC)_5_	5’ GGCTCGTCTCTTCTCTAGCCTCC 3’ 5’ CACTTCTCCAGTCCTGGGGTC 3’	60,4 | 59,3	1181	256	C-terminal Aldehyde dehydrogenase, N-terminal Aldehyde/Histidinol dehydrogenase, cysteine active site, glutamic acid active site
Pg22829	(GCG)_6_	5’ CTCCTCGAATACCTCCCCTTC 3’ 5’ CCTGTCATACGAGTGCCCTAA 3’	61,3 | 60,1	166	166	CCHC-type RNA-binding domain superfamily Zinc finger
Pg24968	(CCT)_5_	5’ TTTCCTTGTCTCACCAGCTCTC 3’ 5’ GAAATCCGATCAGAGGCAAC 3’	60,9 | 59,6	101	101	Isopenicillin N synthase-like SRG1-like
Pg31044	(CTT)_5_	5’ GGAATCCGAAGCAGAAATGG 3’ 5’ GTTGGAGCGAGAGAAGAGGAA 3’	61,8 | 61	134	134	Homeobox-like domain, superfamily SANT/Myb Linker histone H1/H5, domain H15
Pg31517	(CCT)_7_	5’ GCAGCAAAGGAGAGAGAGTGG 3’ 5’ CTATGTTGGACCGGTCTTCGT 3’	61,6 | 61,3	142	142	Auxin-responsive protein, AUX/IAA domain PB1 domain
Pg33230	(CCA)_6_	5’ GCAGCAAAGGAGAGAGAGTGG 3’ 5’ CTATGTTGGACCGGTCTTCGT 3’	61,1 | 61,2	126	126	Leucine-rich repeat domain superfamily Formin-like protein
Pg33733	(TCC)_5_	5’ GGCCAGCCAGTTCAGAATATC 3’ 5’ TTCGGAACAAGACCACTAGGC 3’	61 | 61,5	122	122	Pre-mRNA-processing factor Prp40
Pg34311	(GAA)_7_	5’ GTGTGGAGCCGTTCTTTGAG 3’ 5’ GACCCGTGTCATGGTTCAG 3’	60,8 | 59,9	153	153	Histone deacetylase domain Ureohydrolase domain
Pg35383	(TCT)_6_	5’ ACCTGCTCAGTCGTCGTCTTT 3’ 5’ CCCGTCCAGAAGAACACTCTC 3’	61,4 | 61,2	121	121	Peptidyl-prolyl cis–trans isomerase E RNA recognition motif domain
Pg35926	(GGA)_10_	5’ GAGCTGCTGAACAGCGATTT 3’ 5’ CCACAGATCTCTTCCCCATTC 3’	60,7 | 60,8	158	158	CCT domain
Pg37148	(CGC)_7_	5’ CCCTCCCCATAGTGCTTCTG 3’ 5’ CTCCCTCTCTCCTTCCTTGCT 3’	61,9 | 61,4	107	107	Late embryogenesis abundant protein, LEA_2 subgroup
Pg37450	(GAG)_6_	5’ AAGTAGCCAACGGGCTCAAG 3’ 5’ CCAATTCACTGACGAGAACCTC 3’	61,7 | 61	138	138	Wall-associated receptor kinase, galacturonan-binding domain

## Conclusion

The SSR repeats in coding sequences potentially influence their activity and function, primarily attributed to variations in the repeat length causing phenotypic alterations in the plants. The trimer repeats was the predominant class observed within conserved coding sequences of Psidium guajava. Exploring the polymorphic set of markers, we characterized microsatellites with SRR trimer repeats, showing variation of expression across two important brazilian cultivars. We developed 20 markers related to primary metabolism (Pg16499, Pg19499, Pg34311, Pg35383, Pg37148, Pg37450) and secondary metabolism (Pg33230, Pg14098, Pg20103, Pg20401, Pg33733). This observation suggests the SSR expansion may impact processes related to the maintenance and adaptation of these cultivars, particularly on cellular structures and processes, such as cell membranes and signal recognition, or on stress responses and resistance. The development of markers targeting these specific sites associated with regulatory genes holds potential applications from molecular diagnostics to breeding programs. Also, SSR markers designed for Psidium guajava can be possibly utilized for other closely related species. The triplet SSR sites within these genes present opportunities for evolutionary studies and investigations into methylation process, considering both the repeat characteristics and their GC content.

### Supplementary Information

Supplementary Information 1.

Supplementary Information 2.

Supplementary Information 3.

## Data Availability

The supplementary material with the genes with TR studied were supplied. The transcriptomic data can be obtained through the specified accessions numbers, PRJNA1020439(https://www.ncbi.nlm.nih.gov/sra/PRJNA1020439).Thedatasets generated during and/or analyzed during the current study are available from the corresponding author on reasonable request. Experimental research and field studies on plants (either cultivated or wild), including the collection of plant material, must comply with relevant institutional, national, and international guidelines and legislation*.*
