# Supplementary material for: Effects of trimer repeats on Psidium guajava L. gene expression and prospection of functional microsatellite markers
Source: Sci Rep. 2024 Apr 29;14:9811. doi: 10.1038/s41598-024-60417-8 (PMC11059378; doi:10.1038/s41598-024-60417-8)
Supplement: Supplementary file 1 — Supplementary Information 1. [file 41598_2024_60417_MOESM1_ESM.pdf]

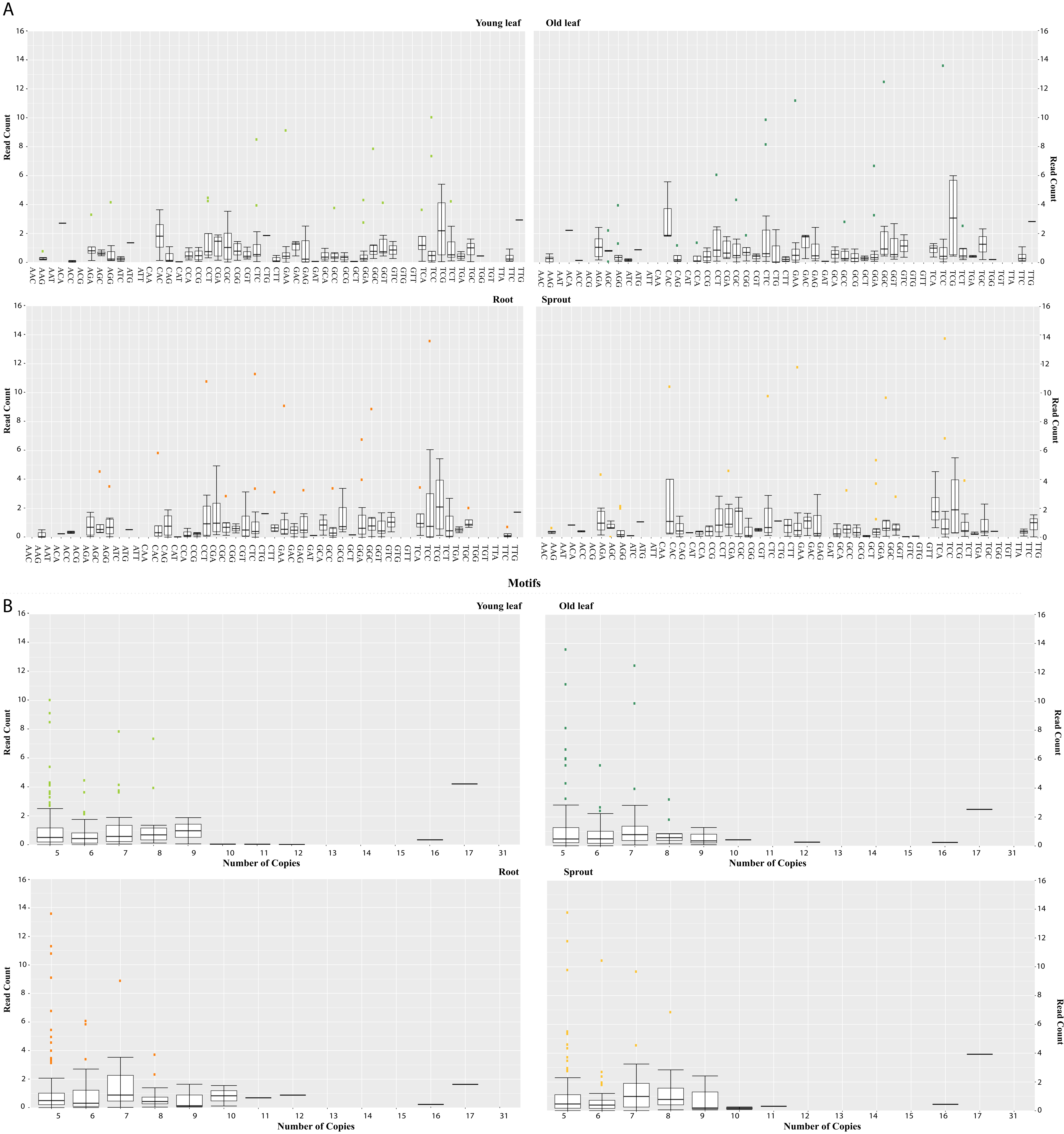

Figure S1. The normalized level of read counts per repeat number and motifs of the 1,107 transcribed genes with TRs from tissues* of Cortibel RM. A) Z-value of all the triplet motifs found in the transcript set. B) Z-value of all repeat numbers found in the dataset.
*Since flower bud RNA was extracted for Cortibel RM alone, such tissue wasn't included in this preliminary analysis for comparison reasons.

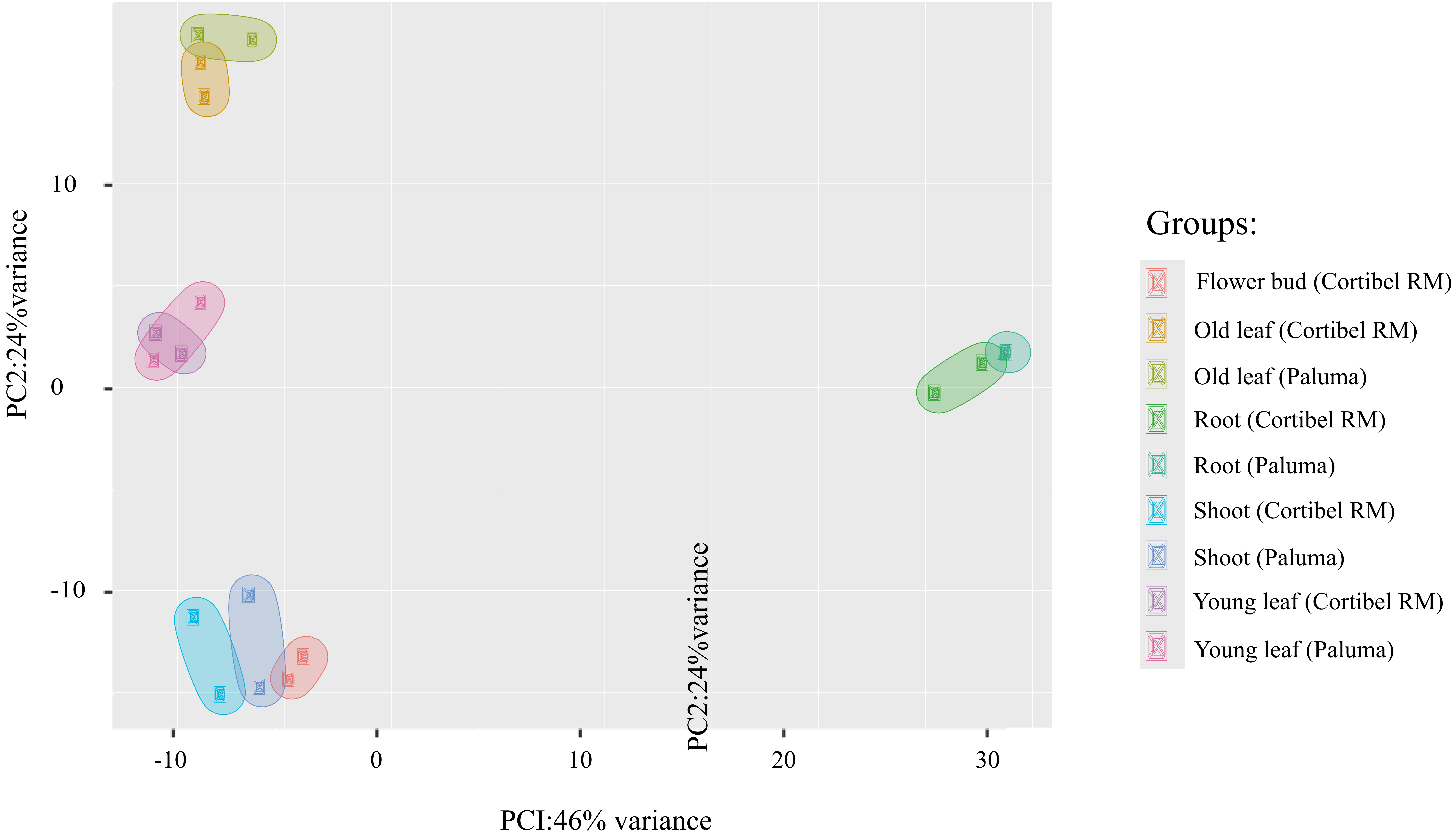

Figure S2. Principal coordinate analysis (PCA) of the normalized read counts of the 1,107 transcribed genes carrying triplet SSRs from the tissues root, shoot, young and old leaves, and flower bud of Cortibel RM and Paluma cultivars.

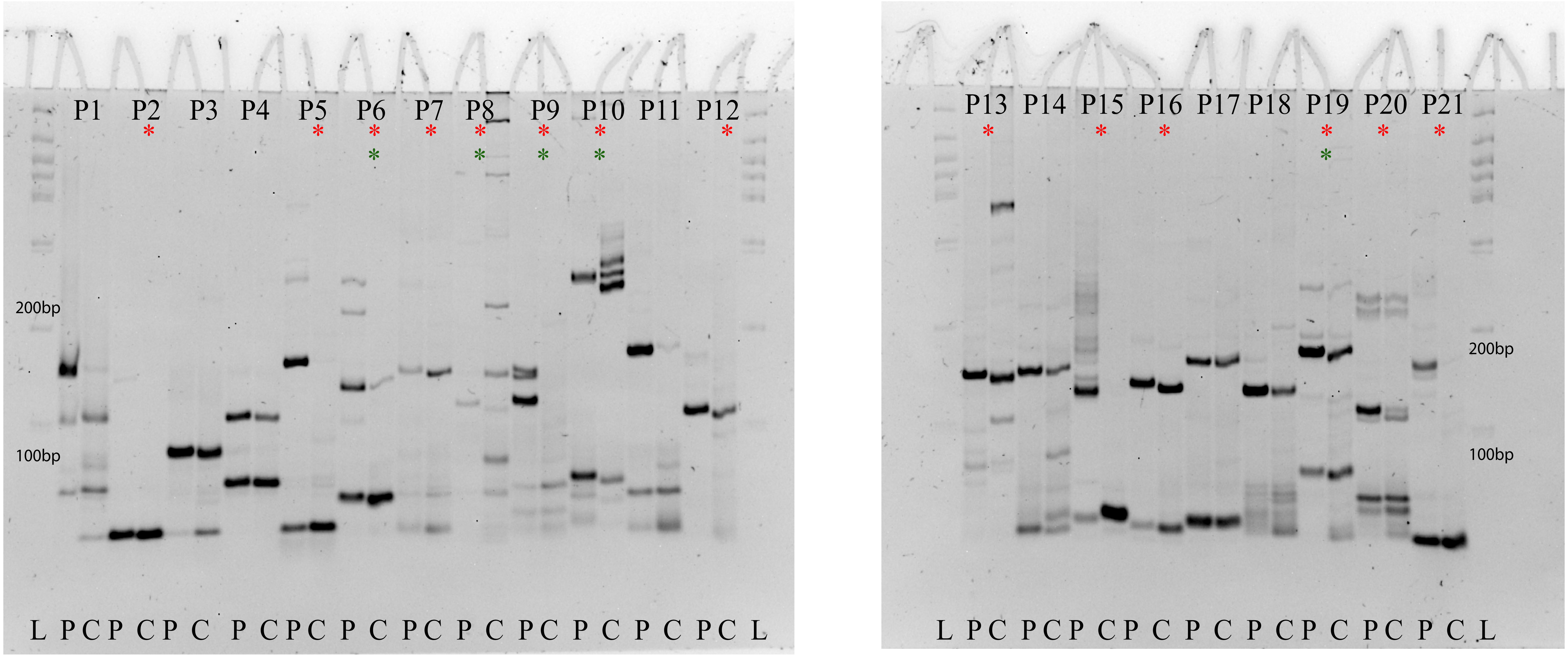

Figure S3. Polyacrylamide gel (10.0%) prepared in TBE buffer of the amplified primers listed in Table 1. Ladder (L) and cultivar Paluma (P) and Cortibel RM (C) are represented. Two µL of each sample were utilized and stained with GelRed diluted at 1:20,000 (Biotium, Fremont-CA, USA).The results were visualized using Gel Doc XR+ software (Bio-Rad, USA). Red asterisks indicate polymorphism between cultivars, while green asterisks represent polymorphism within the same cultivar.
